# Engaging Low-Income Parents in Childhood Obesity Prevention from Start to Finish: A Case Study

**DOI:** 10.1007/s10900-012-9573-9

**Published:** 2012-06-20

**Authors:** Janine M. Jurkowski, Lisa L. Green Mills, Hal A. Lawson, Mary C. Bovenzi, Ronald Quartimon, Kirsten K. Davison

**Affiliations:** 1 Department of Health Policy, Management and Behavior, School of Public Health, University at Albany, State University of New York, 1 University Place, Rensselaer, NY 12144 USA; 2 School of Social Welfare and Department of Educational Administration and Policy Studies, University at Albany, State University of New York, Albany, NY USA; 3 Commission on Economic Opportunity, Troy, NY USA; 4 Department of Nutrition, Harvard School of Public Health, Harvard University, Boston, MA USA

**Keywords:** Community based participatory research, Childhood obesity, Parent engagement, Health promotion

## Abstract

Prevention of childhood obesity is a national priority. Parents influence young children’s healthy lifestyles, so it is paradoxical that obesity interventions focus primarily on children. Evidence and theory suggest that including parents in interventions offers promise for effective childhood obesity prevention. This case study engaged parents’ as co-researchers in the design, implementation and evaluation of an intervention for low-income families with a child enrolled in Head Start. Parent engagement mechanisms include: (1) targeted partnership development (2) operationalizing a Community Advisory Board (CAB) that was the key decision making body; (3) a majority of CAB members were parents who were positioned as experts, and (4) addressing structural barriers to parent participation. Lessons learned are provided for future research, and practice.

## Introduction

Preventing childhood obesity is a national priority for health professionals and policy makers. Consistent with a general call for researchers to engage parents in child health research [1], parental involvement specifically in childhood obesity programs and prevention efforts has been stressed [2–4]. This case study responds to the need for parent engagement as experts throughout the entire research process and, using the example of a childhood obesity prevention initiative, illustrates strategies to engage parents in program development, implementation and evaluation. Parent participation in obesity prevention is increasingly emphasized given links between parents’ attitudes, knowledge, and behavior and children’s dietary, physical activity, and screen-based behavioral factors associated with childhood obesity [5]. Parents are the most knowledgeable about their family’s needs, motivations, and resources for behavioral change, and they understand family dynamics and ecological factors that influence daily living [1]. Parents also have insight regarding program relevance and feasibility. As such, parents active family engagement is crucial for the success of preventive interventions [6, 7].

A growing body of research and relevant theory emphasizes the importance of utilizing parents as change agents in childhood obesity prevention [2, 8]. Although parents have been targeted for studies on treatment of childhood obesity [4, 9], parents are less frequently the direct targets for the prevention of childhood obesity. What is more, the evidence for effective involvement of parents in obesity prevention such as dietary [10] and physical activity [11] interventions is weak. Evidence of program effectiveness among low-income and ethnic minority children who disproportionately experience childhood obesity is also minimal [12]. Parent engagement in research is challenged by low participation rates and high attrition [13]. New approaches are needed to ensure successful engagement of parents in prevention efforts.

**Fig. 1:**
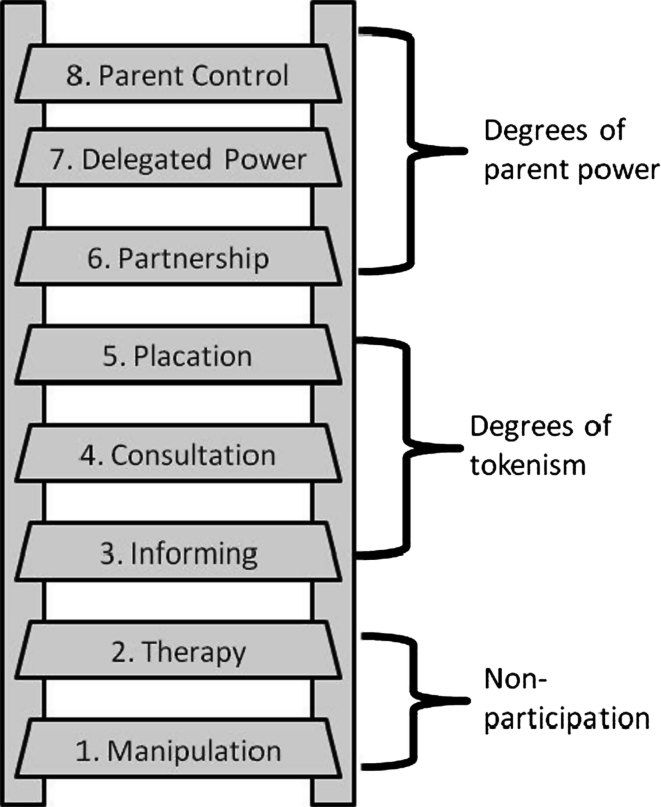
Ladder of Parent Participation. Modified from Sherry Arnstein’s 1969 Ladder of Citizen Participation [14]

One approach is to engage parents in the development, implementation and evaluation of childhood obesity prevention interventions to better integrate parent’s sociocultural context in order to improve program acceptance, cultural relevance and participation. A strategy for operationalizing the level of participation is to utilize the Ladder of Citizen Participation [14], with slight modifications to emphasize the role of parents in health promotion. The Ladder of Parent Participation provides a useful framework for describing the characteristics and extent of parent participation and therefore, the application of CBPR in the literature (See Fig. 1). The ladder has eight rungs representing progressively increasing levels of community engagement. In the case of childhood obesity prevention, high levels of parent participation, in which parents have more contribution to the research process, may improve parent buy-in, participation and program sustainability.

Community-Based Participatory Research (CBPR) is an approach that can be used during the research process to increase the level of parent participation to achieve higher rungs on the Ladder of Participation. CBPR involves community members actively and equitably in decisions throughout the research process, which is often guided by participatory principles [15]. The use of CBPR in childhood obesity research is increasing, but parents, as key stakeholders, are still infrequently engaged. Many CBPR intervention studies to address childhood obesity have primarily engaged community representatives who are in a profession that serves the target population or who have expertise in some area of childhood obesity. Such stakeholders typically include school administrators, teachers, cooks, providers and other community-based professionals [16]. Studies that engage parents, most often fall between Rung 3 and 5 of the Ladder of Participation in which parents provide input and are informed of study processes, often during formative stages of the study, but do not have decision making power. Although other studies have involved parents, there are no known examples in which parents are engaged throughout the *entire* research process. Given the history of hierarchical relationships between low-income families and service or health professionals [17], engaging parents throughout the research process may serve to open communication, break down hierarchical relationships and build trust.

### Case Study Overview

This manuscript describes a parent-centered CBPR case study that expands upon the CBPR literature on childhood obesity prevention by engaging parents directly throughout the entire research process with the goal of fostering parent empowerment and encouraging co-learning across all stakeholders [18]. Low-income parents are engaged as equal partners, providing unique expertise during the development, implementation and evaluation of a childhood obesity prevention initiative. The case study of *Communities for Healthy Living (CHL)*, so named by the partnership, is intended to provide a starting point from which dialogue around engaging parents throughout the research process can begin, propelling the identification of effective engagement strategies that can be tested alongside gains in program effectiveness and sustainability. To this end, we discuss (a) the process of partnership development (Phase 1 of the study), (b) the operation of the advisory board as an effective decision making body, and (c) the provision of structural supports to foster active and equal parent involvement. The conclusion outlines the benefits and challenges of using the CBPR approach to engage parents and lessons learned along the way.

### Research Setting

The *Communities for Healthy Living* case study takes place within the context of a study funded by the National Institute of Minority Health and Health Disparities of NIH, which funded 6 research studies utilizing CBPR in the development of interventions addressing health disparities. Because the studies were funded under the American Recovery and Reinvestment Act of 2009, each was constrained to a rapid 2-year timeline to develop and pilot test the intervention. The goal of this study was to develop and pilot test a childhood obesity intervention for low-income families using a CBPR approach to actively engage parents across three phases, Phase 1: Partnership development, Phase 2: Community assessment and intervention development, and Phase 3: Intervention implementation and evaluation. The family-centered intervention targeted parent/caregivers with children participating in Head Start programs in Rensselaer County, NY (about 500 children ages 6 weeks–5 years old) for childhood obesity prevention. Rensselaer County, in Upstate New York, has areas designated as Medically Underserved Areas [19], and 28 % of all families with children under age 5 living below the poverty level [20].

## Partnership Development

### Formation of the Decision Making Body

A partnership with the community-based organization (CBO) administering Head Start in the county was developed concurrently with proposal development. The CBO director and the Head Start Policy Council, consisting of parents and community members, provided a written commitment to the partnership, feedback on the grant idea, and recommendations for potential community partners. Potential partners were interviewed to determine their interest in the study purpose and their agreement with partner responsibilities. A local reverend of a church serving the neighborhoods where Head Start families reside and a nurse from a local pediatric clinic serving over 60 % of the Head Start families were invited to be partners during this process and became the first members of the planned CHL Community Advisory Board (CAB).

Upon receipt of funding, the Family and Communities Partnership manager for Head Start and program development staff of the CBO were also invited to join the CAB. Candidates for the project coordinator position were jointly interviewed by CBO staff and the research team. Through a subcontract with the CBO, the agreed upon project coordinator was hired as a staff member of the organization. Formally placing the project coordinator within the organizational structure of the CBO was intended to create project visibility at the organization, build relationships with organizational staff and parents, and facilitate organizational cultural exchange. The project coordinator hired had experience working in the community served by the CBO and was responsible for organizing and supporting the CAB, including recruiting additional members to the CAB, particularly parents. It was critical to engage parents early in the process to build trust and foster sustained participation by including them in project decision making as early in the research process as possible with the intent of engaging parents at the highest levels of the Ladder of Participation [14].

CBO staff members on the CAB, who worked directly with Head Start parents, recruited parents of children who currently attended one of the five Head Start Centers and who also exhibited commitment to other Head Start activities. The project coordinator met with the parents to begin the relationship with the project. Additional parents joined after participating in the research or hearing about the project through other parents. Community members recruited to the CAB included a representative from a local cooperative extension, a CBO board member and other community agency representatives who lived within the community and were familiar with community resources. Throughout the first 2 years of the CAB, the board was comprised of 10 parents and 7 community representatives who consistently attended meetings, with several other parents and community representatives attending less frequently. Having parents serve as the majority of decision makers was important for maintaining a high level of parent participation [14]. See Table 1 for composition of the CAB.

**Table 1:** The composition of Community Advisory Board members

Characteristics	N (%)
Head start parent/grandparent	13 (65 %)
Health service professional	2 (10 %)
Community-based or religious organization representative	5 (25 %)
Female	18 (90 %)
Hispanic	3 (15 %)
Black	12 (63 %)
Employed full time	12 (60 %)
Employed part time	3 (20 %)
*Number of children*	
1–2 Children	15 (75 %)
3 or more	3 (15 %)

### Partnership Principles and Operating Guidelines

Many CBPR projects develop principles to help clarify the terms of partnerships, codify expectations between partners and serve as guiding values for the partnership and research process [21, 22]. CHL CAB members reviewed various other CBPR projects’ partnership principles before beginning the process of developing their own during Phase 1 of the project (Partnership Development). The partnership principles were developed during an 8 month period and approved shortly before the end of year 1, although they served to guide CAB activities even prior to final approval. The CAB also decided to create operating guidelines to sustain active involvement, in response to the inconsistent participation of some members. Several CAB members expressed frustration about time spent ‘updating those who do not show up’. A sample of operating guidelines was obtained from a previous participatory project and refined to meet the needs of the CAB. The guidelines were developed, revised and approved by unanimous vote over a 3 month period. The partnership principles and an outline of the operating guidelines are presented in “Appendix A and B”.

## Operation of the Community Advisory Board

### CAB Meeting Structure

Due to the rapid timeline of CHL, CAB meetings were held twice a month for the first 6 months and then once a month for the remainder of the grant. In total, 25 meetings, including Workgroup meetings were conducted during the study. These meetings were held in one of the CBO’s buildings housing a Head Start center. Agenda items for the meetings were created with input from the academic staff, CBO staff, the project coordinator and CAB members. The meeting structure varied depending on agenda items, and included a combination of small group and whole group discussions. Meetings were primarily run by the project coordinator, with the researchers facilitating when there was discussion and interpretation of data, and CAB members leading discussion of specific agenda items. Although efforts were made by the project coordinator to have a formal leadership structure within the CAB, none of the CAB members wanted to be an officer.

### Small Work Group Meetings

Full CAB meetings were supplemented throughout the 2-year project with small Work Group meetings held at the CBO and at the university. During the first 3 months, smaller parent only meetings were held prior to full CAB meetings to foster social connections among parents. Discussions in these groups during Phase 1 focused on encouraging parents to think critically about factors that influence children’s risk for obesity and to participate as experts and co-researchers. These meetings provided time for parents to talk openly about their experiences as parents and to ask questions without CAB professionals present. After three of these meetings, parents felt comfortable being vocal in the larger CAB. By the fourth month of CAB meetings, parents had a strong presence at meetings and were active participants in the research process.

The full CAB was also split into four small Workgroups to focus on multiple aspects of the research simultaneously. Most of the CAB participated in at least one group but some CAB members chose to participate in multiple groups. An Ethics Workgroup focused on the participatory process. A Data Workgroup helped guide the community assessment by developing the focus groups’ topic and interview guide, conducting data analysis and interpreting findings. An Education workgroup guided the development of materials for the Parents Connect for Healthy Families curriculum. A Social Marketing Workgroup developed the *Communities for Healthy Living* logo, mission, project pamphlet and childhood obesity awareness poster campaign. All of these features were important for branding and were included in communications, and CHL sponsored events.

## Fostering Active Participation of the Community Advisory Board

### Active Engagement Throughout the Research Process

**Fig. 2:**
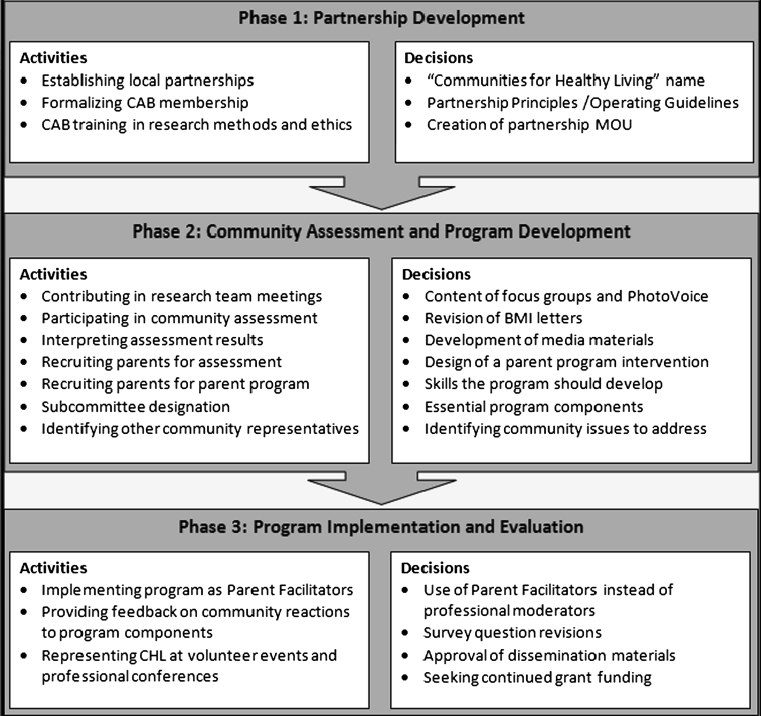
Community Advisory Board Parent involvement in communities for Healthy Living activities and decisions throughout the 3 phases of the project

Although it is not unusual to have advisory boards on which community members provide input but do not share decision making power, this study’s aim was to involve CAB parents at rung 6 or 7 of the levels of the Ladder of Participation (Fig. 1); therefore, there was a need to foster CAB involvement outside of CAB meetings. A project policy was to include CAB members in as many activities as they were willing to participate. In addition to participating in CAB meetings, parents participated in day to day research activities alongside academic partners as equal partners. Their expertise was highly valued and included when decisions were made for the research activities. Figure 2 presents a summary of CAB activities and decisions, which varied across the three phases of the project. During Phase 1 of the project, the main focus was partnership development. In Phase 2, the CAB fully participated in a thorough community assessment and the design of the Communities for Healthy Living intervention. In Phase 3, the CAB focused its efforts on program implementation and evaluation.

The first CAB meeting during *Phase 1* was essential for setting the participatory tone and describing the purpose of the funded research. Academic staff described the specific aims of the project including the CBPR approach, the role of parents as experts, the responsibility to the funder and what is known about childhood obesity and its risk factors with parents. At that point, the project coordinator engaged parents and community members in a discussion to obtain preliminary perspectives on childhood obesity. During the second and third meetings, the CAB worked in small groups with a flip chart and a set of questions to discuss. They were asked to prioritize the essential barriers and facilitators to child health, family health, and parents’ ability to take care of their children’s health. Benefits of this process include, (1) increasing critical consciousness (a component of empowerment) of childhood obesity among CAB members, (2) identifying social determinants of childhood obesity and other child health issues that were relevant to their community, (3) building relationships between CAB members and the CHL academic staff, and (4) operationalizing the expertise of parents by documenting their contribution to these discussions. During these meetings, CAB members were also trained in research ethics and received IRB certification. Phase 1 of Fig. 2 outlines the specific activities in which CAB members participated and the decisions in which they were actively involved.

During *Phase 2* of the project (Community assessment and Program Development), CAB parents participated in the design and implementation of the mixed-method community assessment, the dissemination of the results, the development and implementation of the intervention and its evaluation. CAB member participation in research team meetings during Phase 2 facilitated their participation in decision making on a continual basis equal to that of research team members. They suggested that certain discussions needed to be brought to the entire CAB and they were involved in project problem solving and data collection planning. Research team members, parents, and other CAB members worked together to develop research questions and develop and revise data collection instruments. Several parents also recruited and administered assessment tools.

Also during Phase 2, some CAB parents spent their summer integrally involved in intervention development (see Phase 2 Decisions in Fig. 2). In addition to being involved in the step by step design of the social marketing campaign and other educational material targeting parents, they also helped develop a 6-week parent program, *Parents Connect for Healthy Families,* and an intensive 4-day train-the-trainer session for parent facilitators. The program focused on increasing awareness of childhood obesity and its risk behaviors and providing communication, conflict resolution, stress management, and social networking skills, including how to leverage community resources.

During *Phase 3* of the project (Program Implementation and Evaluation), four of the CAB parents participated as program facilitators. These Head Start parents participated in a 4-day training seminar along with other parents and then facilitated the administration of the *Parents Connect for Healthy Families* curriculum to their peers in the Head Start community. Engaging parents in both the design and leadership of the program ensured its relevance, and was an important part of the participatory process. Other parents who joined the project as program facilitators subsequently joined the CAB after their experience working with the program.

### Structural Support for Parent Engagement

Several structural supports were put in place to encourage consistent parent engagement. With the exception of data analysis and research meetings, CAB and most Workgroup meetings were held at a Head Start center immediately after the end of the school day. Parents were able to pick their children up and attend meetings in the same building. Childcare was provided onsite by Head Start teachers. Dinner was also provided to CAB members and their children at the beginning of CAB meetings, which allowed time for free conversation. This opportunity for community representatives, parents and university staff to interact helped build relationships. CAB members networked with each other and the academic staff, which led to tangible benefits for many members. Examples include a parent talking to a nurse about her interest in becoming a nurse, and another talking to the researchers about programs offered at the university.

Finally, parent engagement was encouraged by the provision of gift cards. Members of the research team were compensated by the grant. To reinforce the stated value of equality, CAB members were offered $25 gift cards to acknowledge the time and expertise they contributed. CHL also offered gift cards for parents who volunteered in activities such as recruitment, data interpretation, and intervention development and facilitation. While they were warmly received by the parents, several parents expressed that although the cards were helpful, they would still attend meetings if they were not offered because they are committed to the project.

**Fig. 3:**
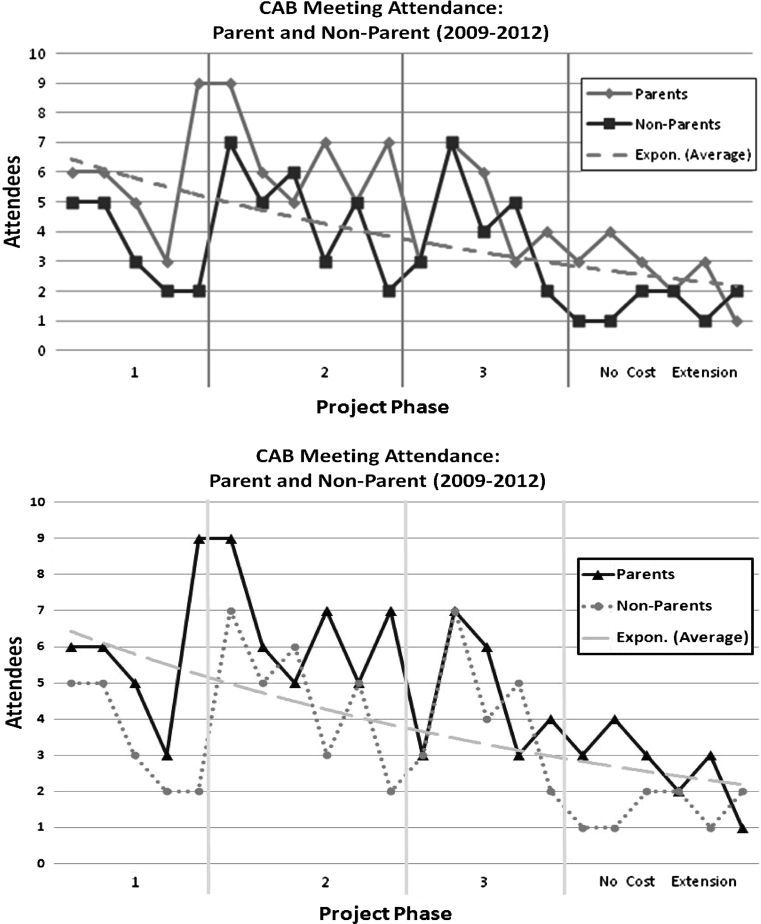
Community Advisory Board attendance throughout the 3 phases of the communities for Healthy Living Project

Although a core group of CAB parents and community members participated across all project phases, CAB attendance decreased over time as the project had fewer decisions to make. This is obvious in Fig. 3 showing meeting attendance throughout the project phases. During Phase 3 the focus shifted to program implementation and the majority of CAB parent involvement shifted towards participating as a parent facilitator or by helping the project coordinator administer the parent program or social marketing campaign. After the completion of the pilot intervention, the project focused on the evaluation, including data entry and analysis. Fewer parents attended meetings as there was less to do until the data was ready to present. However, four to five parents participated in data entry and other research activities during this time. One parent attended two conferences and presented on CHL alongside researchers. Also, parents continue to participate in the development of abstracts, posters and presentations for dissemination of the results. They are also actively involved in the development of additional research grant proposals.

## Discussion

### Summary of Parent Participation

The research team employed various innovative strategies and structural accommodations which successfully fostered parents’ continuous involvement in decision making and day to day activities *throughout* all phases of the research process as ‘experts’, hence engaging CAB parents at the highest rungs of the Ladder of Participation. Parents were equal to the researchers and community representatives, whose roles on the CAB were related to their professions. Parents engaged in co-learning with community members on the CAB and academic staff, sharing their expertise, a necessity in child health research [1]. Most previous childhood obesity interventions [16, 23] involved parents or caregivers at the level of informed consultant (the fifth rung of the Ladder of Participation) which involves community members as advisors, whose input may or may not influence decisions [14]. These studies, [16, 23] advanced the field in that parents were involved in the intervention development process, during which parents gave input and advice and were informed how their input influenced the subsequent intervention. However, CHL is the only known study that achieved the highest rungs of the Ladder of Participation in which parents participated throughout the entire research process.

### Benefits

There were many intended and unintended benefits gained as a result of this study’s CBPR approach. Parents displayed strong buy-into CHL’s messages and activities and on their own accord, promoted the CHL intervention to other Head Start parents and organization staff. These strategies resulted in sustained active participation of parents that led to additional trained, committed co-researchers that (a) contributed unique and valuable expertise to the project and (b) resulted in a more salient, culturally-responsive and sustainable intervention.

Although the purpose of this paper is to describe rather than evaluate the participatory process (evaluation presented elsewhere) [24], the benefits to parents were identified anecdotally and through CAB evaluation surveys and in-depth interviews. Briefly, parents expressed that they built supportive relationships with each other. The co-learning among parents and between parents, academic staff and community organizations influenced parents’ knowledge of resources as well as their confidence to access and utilize those resources. For example, at least two of the ten CAB parents decided to pursue an academic degree after speaking with other parents with young children who recently completed programs. One parent who completed college while her child was in the Head Start program mentored another parent to help her learn study skills. Many parents reported adding skills they learned through CHL to their resume. One reported at a CAB meeting that adding the skill of interviewing helped her get a new job. Analysis of in-depth CAB member interviews found that parents described an increase in knowledge and confidence about their ability to advocate and disseminate their knowledge within their community. The evaluation of the participatory process will be presented in a separate paper.

### Challenges

The level of engagement for parents resulted in some repercussions. First, some of the leadership of the partner organization were concerned that empowering parents through active engagement may create activist parents who would become vocal with local politicians using the community organization’s name. They feared the potential creation of rifts that the organization could not afford. The researchers responded by promising to appropriately train parents if they decided to advocate outside the organization and reminding parents that there is a protocol to follow for speaking on behalf of an organization.

Some community/organizational representatives felt unclear about their role on the CAB because of the focus on engaging parents. This resulted in inconsistent participation among some. Regardless, a core group of community representatives participated regularly and gave positive feedback on the role of parents and the benefits of participation. In addition, non-parent CAB members tended to re-engage during the second year of the study as the intervention began. Of the organizational representatives who participated in the first CAB meeting, all but one were still involved in the project and attending CAB meetings in the second year of the study.

Additionally, there was the perception of the development of a hierarchy among some parents, during Phase 2, the implementation of the childhood obesity intervention targeting all Head Start families. It was expressed that parents who were parent program facilitators developed stronger relationships with each other and the academic staff as a result of their greater level of participation. It is notable that the parents who felt this hierarchy felt comfortable expressing their feelings to the project coordinator. The coordinator made extra effort to reconnect with parents whose participation dropped off in response. Another challenge was the level of parent expectation of what CHL staff would actually be able to do for them. At times, CHL staff may have been perceived as service providers similar to staff at the CBO. While CHL staff were supportive of parents, there were limitations to how CHL staff could assist parents. Some parents initially expressed frustration, but through on-going discussions and role clarification, they became comfortable with the level of support provided.

Finally, formalizing and sustaining the 17 member CAB was a challenge during Phase 3 and the no cost extension of the grant. After two attempts to have an election of CAB officers, the idea of creating a formal CAB with officers never came to fruition. CAB members had multiple competing priorities and although they were actively involved in CHL, they did not want to commit to running meetings or potentially delay activities and decisions if enough of the officers did not attend a particular meeting. Further, during Phase 3, CAB member participation dropped to a core group of nine members and during the no cost phase of CHL, CAB meetings had an average of four members. Although these members are active as described by their participation in dissemination and grant proposal development, maintaining the CAB without an active intervention research agenda poses a challenge.

### Lessons Learned

This case study identified specific strategies to foster parent engagement. Structured by a commitment to engage parents as true experts and equal partners, the participatory process was careful to build skills and facilitate consistent and active participation so that parents were able to be equal partners in the research process. The use of small groups helped foster confidence among parents as well as allowed CHL staff to emphasize their commitment to parents being considered valuable experts. The implementation of planned, focused activities and designated networking time over meals fostered interaction above and beyond project conversations and fostered trust, which was important for relationship building and a positive work environment in the CAB. The development of operational guidelines and partnership principles set the tone for the level of commitment needed, created a mission for the CAB, and maintained the infrastructure of parent involvement. Placing the project coordinator at the community organization and hiring one that was familiar with the neighborhoods served by the Head Start Centers was essential for cutting across the community and academic cultures and also represented a commitment to the community and community outreach. The structural support of meals, incentives, child care and convenient meeting locations not only demonstrated the commitment to parent involvement but also facilitated parent involvement as shown by the level of participation (see Fig. 3). All of the aforementioned encouraged involvement of parents throughout the entire research process.

## Conclusion

CHL’s successful engagement of parents in the design, implementation and evaluation of an intervention to address childhood obesity adds to the childhood obesity intervention literature. The outlined CBPR strategies to facilitate parent engagement were designed to avoid tokenism [15]. CHL’s innovative design of engaging parents as “experts” successfully bridged the cultural, socio-economic, and interpersonal divides between parents and the professionals which resulted in a true participatory process. Leveling the playing field in research with low-income parents is more challenging than doing so with community organization representatives because of the lack of education or traditional forms of expertise defined by employment or a profession. CHL brought together people with different levels of privilege to work as equals on a research project. The challenges of treating and engaging parents typically known as “clients” and “the target population” as equal members on the CAB should not be under-estimated. CHL was designed to address this challenge, and the strategies used in CHL can inform other CBPR studies.

To advance the field and improve child health, it is essential to work with parents in the research process. By documenting CHL’s participatory process and concrete strategies for engaging parents, other child health researchers should be encouraged and empowered to actively engage parents and other caregivers in their research, which will in turn benefit the health of children and families. The strategies described in this case study are examples of strategies that other researchers can use to engage parents in the research process. All of CHL’s strategies have a fundamental underlying point of view: parents can be engaged as experts in child health research and their expertise is valuable and essential. From this vantage point, other researchers can also employ these strategies, all for the benefit of (1) childhood obesity research and (2) most importantly, the “target population”, families who have children at risk for or who experience this growing public health problem.

## Appendix A

See Table 2.

**Table 2:** Communities for Healthy Living partnership principles

(1) The identity and uniqueness of the community must inform the research and the research must inform the community *Expectations* (a) Identify the unique assets, resources, and needs of the community to promote healthy lifestyles. (b) The participatory assessment must be a foundation for the intervention (c) The identity of the community must be acknowledged in the interpretation and dissemination of the assessment
(2) The CAB, CEO partners, and university staff are committed to a research process in which each step informs the next step of the project and the work leads to action *Expectations* (a) In order for this principle to be implemented, first and foremost, confidentiality and integrity of participants must be protected (b) Data must be shared with the CAB, CEO partners and the community (c) Findings of data must be shared with the community in a language that they are able to understand and that is culturally sensitive (d) Must have a vehicle to communicate information to the community and to collect information from the community by reaching out to families where they are (e) The CAB, CEO partners and university staff must be accountable to the community
(3) Communities for Healthy Living activities allow for active and equal involvement of CAB members and university staff *Expectations* (a) CHL is a partnership, so all members should be able to depend on each other to move the goals of CHL forward (b) University staff will provide all information and sufficient training to engage the community
(4) Communities for Healthy Living builds upon the strengths and expertise of CEO, CAB members and University at Albany staff *Expectations* (a) All members should learn from each other (b) Project strategies and programs will reflect the expertise of all member groups
(5) The research process empowers people to understand the many factors that influence the health of CEO Head Start families and act upon those factors to promote health *Expectations* (a) To promote a healthy lifestyle and address childhood obesity, people will gain knowledge about healthy lifestyles and strategies for having a healthy lifestyles through participation in CHL

## Appendix B

See Table 3.

**Table 3:** Communities for Healthy Living operating guidelines

Ethical responsibilities of board members to the research process *Respect for each other—members agree to* Regularly attend meetings of the Community Advisory Board and its sub-committees Arrive to meetings at the agreed upon arrival time Contact the project coordinator if going to be absent or late for meetings To review documentation provided at meetings in preparation for participation in discussion at subsequent meetings Respond to the project coordinator or other members of the research team either via phone or email within at least 48 h of any request for information Focus on problem-solving dialogues and conversations Recognize conflict and diversity as assets to be maximized, not problems Develop conflict resolution procedures, which will address how matters will be resolved including actions requiring a vote
*Respect for the research process—members agree to* Respect each others’ confidentiality Use a democratic process and reach a preliminary consensus on CAB decisions Remain open to comments from all CAB members, and work toward a process that ensures that all members’ voices are heard Maximize the time set aside for CAB meetings by limiting distractions and developing a prioritizing process that is acceptable to all/majority of members Develop an orientation/mentoring process for members joining late in the process and for members who miss a meeting, ex. “buddy system” Work towards barrier-busting and problem-solving mechanisms
*Respect for the community—members agree to* Keep the focus on the needs, problems, interest and aspirations of the children and families at the center of our work, remembering that they are the reason for our individual and collective efforts Not make “turf” an issue and will seek shared ways to problem solve, divide workloads and modify roles and responsibilities to meet the needs of the project Use strength-based, solution focused, culturally responsive language Share power, responsibility and knowledge Build from and honor collaborative agreements among individuals and inter-agency organizational commitments and agreements, provided any collaboration entered into on behalf of the CAB has been openly discussed and agreed to by a consensus of CAB members
